# The Sex and Race/Ethnicity-Specific Relationships of Abdominal Fat Distribution and Anthropometric Indices in US Adults

**DOI:** 10.3390/ijerph192315521

**Published:** 2022-11-23

**Authors:** Furong Xu, Jacob E. Earp, Alessandra Adami, Ingrid E. Lofgren, Matthew J. Delmonico, Geoffrey W. Greene, Deborah Riebe

**Affiliations:** 1 School of Education, University of Rhode Island, 142 Flagg Road, Kingston, RI 02881, USA; 2 Department of Kinesiology, University of Connecticut, Storrs, CT 06269, USA; 3 Department of Kinesiology, University of Rhode Island, Independence Square, Kingston, RI 02881, USA; 4 Department of Nutrition and Food Sciences, University of Rhode Island, Fogarty Hall, Kingston, RI 02881, USA

**Keywords:** anthropometric indices, body mass index, waist circumference, visceral fat area, subcutaneous fat area, visceral to subcutaneous fat area ratio, health disparities

## Abstract

The purpose of this study was to examine demographic-specific relationships between direct abdominal fat measures and anthropometric indices. A cross-sectional study was conducted utilizing abdominal fat measures (visceral fat area, VFA; visceral to subcutaneous adipose area ratio, VSR) and anthropometrics (body mass index, BMI; waist circumference, WC) data from the 2011–2018 National Health and Nutrition Examination Survey. Linear or polynomial linear regression models were used to examine the relationships of abdominal fat measures to anthropometrics with adjustment for demographics. The results revealed that while VFA was linearly related to BMI and WC across all demographics (*p* < 0.001), the relationships between VSR and both BMI and WC were concave in men and convex in women. The relationships between VFA, VSR, and BMI, WC varied by sex and race/ethnicity. In conclusion, increasing BMI and WC were linearly associated with increased VFA, but their relationships with VSR were nonlinear and differed by sex.

## 1. Introduction

The rising obesity epidemic in the US is a national health concern due to the increased health risks related to excess body fat [1]. Body mass index (BMI) and waist circumference (WC) are common anthropometric indices for obesity-related health risks due to their positive relationships with body fat and regional body fat distribution [2,3,4,5,6]. These parameters are more practical, efficient, and economically sound when compared to direct measurements of body fat, such as dual-energy X-ray absorptiometry (DXA) [7], which is a two-dimensional imaging technique to assess total and regional adipose tissue mass and distribution. However, BMI is not a measure of the total percentage of body fat or abdominal adiposity because it cannot distinguish between lean, fat mass and does not consider adipose tissue distribution [8]. Similarly, measurements of WC do not account for the overall body size or shape [9] and thus may misrepresent body adipose tissue concentration and distribution [10,11,12]. Therefore, it is important to better understand the relationships between BMI and WC with direct measures of overall and abdominal body fat [13].

Although many studies have examined the relationships between BMI and WC with body fat percentage [2,3,4], the relationships between BMI and WC with abdominal fat measures have received less attention, and studies present varied results [5,14,15,16,17,18]. Of these, studies that have focused on a single population have reported positive relationships between BMI and/or WC with the visceral fat percentage in young Thailanders [14], with the visceral fat mass in White adults [6,15], and with the visceral fat area (VFA) in Indian adults [16]. Additionally, several studies have considered racial/ethnic differences in the relationships between BMI and WC with VFA [5,17,18] with inconsistent results. Specifically, one study reported no significant race/ethnicity effects between BMI and VFA in adults aged 40–64 years [5], while the other two reported that White men and women have higher VFA than Black and/or Hispanic men and women of the same BMI and WC [17,18]. However, the previous studies on specific race/ethnicity relationships between BMI or WC with visceral adipose were often studied primarily among White and Black populations [5,17], with only one study including Hispanics [18] and none including Asian and other population groups [5,17,18]. In addition, research about the relationships between BMI, WC, and visceral-to-subcutaneous adipose area ratio (VSR) is limited, even though VSR is an independent predictor of cardiometabolic risks [19,20,21]. We identified only one study that focused on the relationship between BMI, WC and VSR using a sample from the New England area of the US [19]. Thus, it is necessary to expand on previous studies and further examine the relationships between BMI and WC with abdominal indices such as VFA and VSR using a nationally representative sample of US adults measured using standardized anthropometric procedures and DXA. Accordingly, the present study aims to address the above research gaps using a nationally representative adult sample. The objective of the present study is to assess the sex and racial/ethnicity-specific relationships between conventional anthropometric measures [BMI and WC] with VFA and VSR.

## 2. Method

The present study is a secondary data analysis of the National Health and Nutrition Examination Survey (NHANES) 2011–2018 data [22]. Respondents aged 20 years or older who had demographic, VFA and subcutaneous fat area data were included in this study. Accordingly, of a total of 14,934 respondents aged 20 years or older, 707 were excluded due to missing WC and/or height or weight data, and 2284 were further excluded due to missing visceral fat-related or subcutaneous fat area data. The final analysis included 11,943 respondents aged 20–59 years who fulfilled all inclusion criteria. This study has been approved by the University of Rhode Island Institutional Review Board (IRB) (IRB# 1945004-1).

### 2.1. Demographics

Standard NHANES demographic variables were utilized to report sample characteristics, including age, sex (male vs. female), race/ethnicity, education, and the ratio of family income to poverty [22,23]. For the current study, the age was categorized as 20–39 years or 40–59 years; race/ethnicity was categorized as Black, White, Hispanic (Mexican American and other Hispanic), Asian and others; education was classified into two categories (high school or less and some college or more); and the ratio of family income to poverty was classified into two categories (<1, below the poverty line; and ≥1 at or above poverty line) from numerical data (0–4.99 or 5 or more) as previous studies did [24].

### 2.2. Anthropometrics

Height (cm), weight (kg), and WC (cm) were measured at NHANES Mobile Examination Center by trained health technicians and followed standardized protocols described previously in detail [22,23]. BMI was calculated using measured height and weight and was further classified into underweight (BMI < 18.5 kg/m^2^), normal (18.5 kg/m^2^ ≤ BMI ≤ 24.9 kg/m^2^), overweight (25.0 kg/m^2^ ≤ BMI ≤ 29.9 kg/m^2^) and obese (30.0 kg/m^2^ ≤ BMI) according to Centers for Disease Control and Prevention recommendations for adults 20 years or older [25]. Abdominal obesity was classified according to sex-specific WC classifications (males, ≥102 cm; females, ≥88 cm) [26].

### 2.3. Abdominal Fat Distribution

Two measures related to abdominal fat distribution were considered: (1) VFA, the amount of fat (cm^2^) located inside the abdominal cavity, around the internal organs of the abdomen or the abdominal walls, was measured using DXA [23]; (2) VSR, calculated by dividing VFA by the subcutaneous fat area, which is the amount of fat (cm^2^) located directly beneath the skin, was also measured using DXA [23,27].

### 2.4. Data Analysis

Given the complexity of the survey design, the combination of 8-year Mobile Examination Center exam weight was constructed and then utilized for all data analyses according to NHANES’s data analysis guidelines [28]. All descriptive data were summarized by using weighted means and standard errors or counts, and weighted percentages for all study participants. Continuous variables were compared using the *t*-test (PROC SURVEYREG procedure), and the Chi-square test was used to compare categorical variables (PROC SURVEYFREQ procedure). A linear regression model and a polynomial linear regression model with a quadratic term if the trend indicated a non-linearity were used to estimate the association of BMI and WC with the level of VFA and VSR, separated by sex and race/ethnicity. Then, interaction terms (BMI × race or WC × race) were added to the model to further explore the race/ethnicity effect on the relationships. All of the models were adjusted for age, sex, race/ethnicity, education, and the ratio of family income to poverty. It is important to note that race/ethnicity was only adjusted in the total and the non-race/ethnicity-specific analyses. Statistical significance was defined as *p* < 0.05. Analyses were performed with SAS version 9.4 (SAS Institute Inc., Cary, NC, USA).

## 3. Results

**Figure 1:**
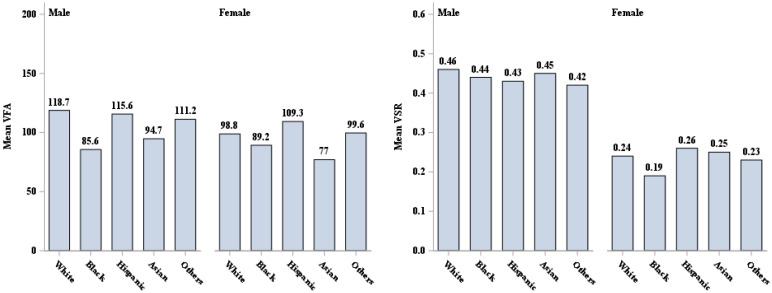
VFA (cm^2^) and VSR description by sex and race/ethnicity. VFA = visceral fat area, VSR = visceral to subcutaneous adipose area ratio.

**Figure 2:**
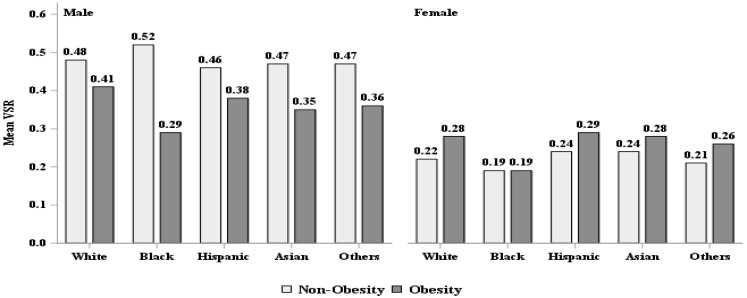
VFA (cm^2^) and VSR description by body mass index classified obesity. VFA = visceral fat area, VSR = visceral to subcutaneous adipose area ratio.

**Figure 3:**
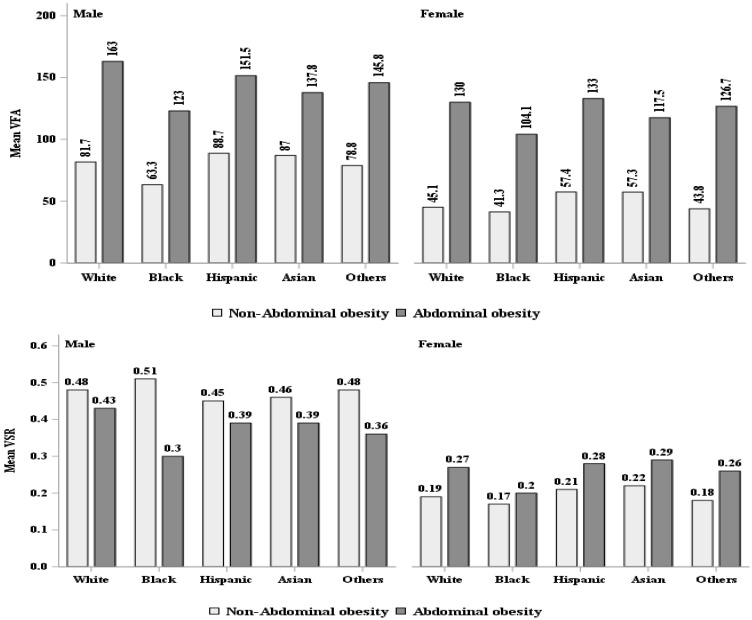
VFA (cm^2^) and VSR description by waist circumference classified abdominal obesity. VFA = visceral fat area, VSR = visceral to subcutaneous adipose area ratio.

Approximately half of the sample was female (48.4%); 38.3% were racial/ethnic minorities, with 11.8% Black, 17.0% Hispanic and 5.8% Asian; 34.7% had a high school education or less, and 15.8% had a family income below the federal poverty level. Nearly 70% of the sample were either overweight (32.0%) or obese (37.6%), as defined by established BMI cutoffs. Over 50% of the sample had abdominal obesity (52.6%), as defined by WC cutoffs. Males were more likely to be overweight or have higher WC, VAF and/or VSR than females, whereas more females were classified as having abdominal obesity (Table 1). The pattern for VAF and VSR between males and females appeared to be similar across sex-specific racial/ethnic groups (Figure 1). All individuals with obesity or abdominal obesity appeared to have higher VFA compared to those who were not obese, regardless of sex. However, our findings showed that males who were not obese and without abdominal obesity had a consistently higher VSR than those who were obese or who had abdominal obesity in all male-specific racial/ethnic groups (Table 1, Figure 2 and Figure 3).

**Figure 4:**
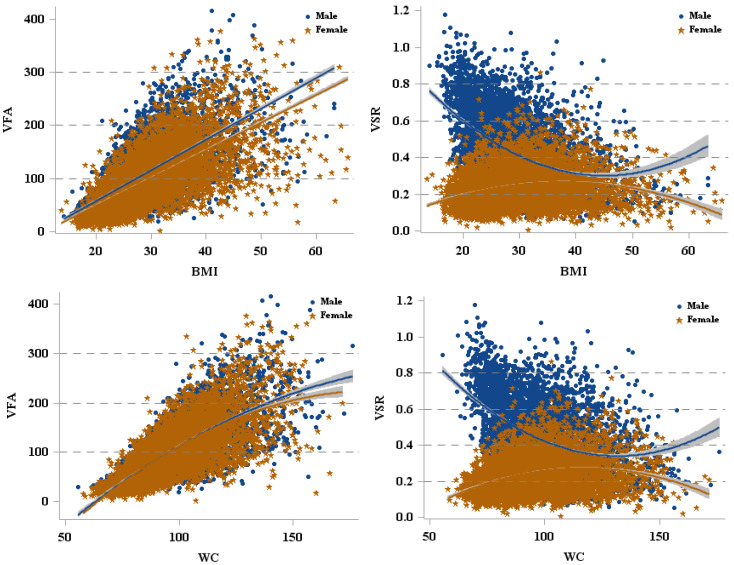
The relationships between VFA and VSR with BMI, WC. VFA = visceral fat area, VSR = visceral to subcutaneous adipose area ratio, BMI = body mass index, WC = waist circumference.

The analyses revealed linear relationships between BMI and VFA or WC and VFA (Figure 4). More specifically, after adjustment for demographic variables, BMI was positively associated with VFA (β = 5.86, 95% CI: 5.56, 6.15, r^2^ = 0.609, *p* < 0.001). A similar pattern was observed across different racial/ethnic groups, male and female groups, and sex-specific racial/ethnic groups (Table 2). Moreover, the adjusted analysis also revealed a positive relationship between WC with VFA (β = 2.64, 95% CI: 2.53, 2.75, r^2^ = 0.684, *p* < 0.001). This positive relationship was also observed in males and females and all racial/ethnic groups or sex-specific racial/ethnic groups (Table 2). There were race/ethnic group differences in BMI and WC relationships with VFA, with the White group significantly different from the Black and Hispanic and/or Asian groups (Table 2).

There was either a concave (male) or convex curvilinear (female) relationship between BMI and WC with VSR (Figure 4). In addition, due to the opposite relationships between BMI and VSR, WC and VSR, we reported the results for males and females separately (Figure 4). In males, when BMI < 39.08 kg/m^2^, BMI was negatively associated with VSR; however, when BMI ≥ 39.08 kg/m^2^, the relationship between these two variables was reversed, with a higher BMI associated with a higher VSR. In females, BMI was positively associated with VSR until it reached 38.75 kg/m^2^ but was inversely associated with VSR for BMIs ≥ 38.75 kg/m^2^. A similar pattern was observed between WC and VSR in males and females. Furthermore, the relationships between BMI or WC and VSR were different in racial/ethnic groups, with Black significantly different from White, Hispanic, and Asian (Table 3).

## 4. Discussion

The present study extends previous work that describes the relationships between anthropometric measures and the distribution of abdominal fat. To the best of our knowledge, we are the first to examine such a relationship using a large, representative sample of the adult US population accounting for its diversity, expressed in terms of sex and race/ethnicity. Our findings better represent the influence of the difference in population characteristics compared with previous studies that focused on smaller samples, primarily of Black or White non-Hispanic individuals [5,14,15,16,17]. Our findings revealed consistent positive relationships between VFA and both BMI and WC in both sexes and all racial/ethnic groups. Interestingly, there were patterns of opposing relationships between VSR and both BMI and WC between the sexes, with a concave curvilinear relationship observed in men and a convex curvilinear relationship observed in women. Furthermore, we found racial/ethnic group differences in the relationships between VFA and VSR with both BMI and WC.

The current study’s findings on the relationships between VFA and both BMI and WC were consistent with previous studies [5,17,18]. We observed that independent of age, education or family income, the relationships between VFA and both BMI and WC varied by race/ethnicity. Of note, the estimation of the slope parameter for White people was higher than for people with Black, Hispanic and/or Asian race/ethnicity classification, whereas the slope parameter for Black people was lower than for White, Hispanic, and Asian people. These findings indicate that with similar BMI or WC, White people will likely have higher VFA, whereas Black people have lower VFA than other racial/ethnic groups. This phenomenon was also observed in our sex-specific racial/ethnic group analyses. The only exception was the relationship between WC and VFA in women, in which the slopes for WC and VFA were significantly higher in White women than in Hispanic women but lower in Black women than in Hispanic and Asian women. These findings are somewhat consistent with previous research on the results for Black men and women [17,18,29] and for White men and women [5,18,30]. However, our analysis extends the results of previous studies because we focused on a larger sample of the general US adult populations, including more racially diverse samples of Hispanic and Asian people. A possible explanation for the higher positive correlation between BMI and WC and VFA in White compared to Black samples could be a difference in race-related sex-hormone-binding globulin and testosterone concentrations [31], which might result in a difference in visceral adipose accumulation. However, the cross-section study design of the study limits our ability to explore the causality, and further studies are warranted to examine the possible reasons for relationship differences by race/ethnicity.

We also examined the sex-specific racial/ethnic relationships between VSR and both BMI and WC. VSR provided the relative abdominal fat composition, which is important since visceral fat has known stronger relationships with cardiometabolic risks rather than subcutaneous fat [32,33]. Our findings are interesting as there were opposing concave (male) or convex (female) shaped curvilinear relationships between VSR and both BMI and WC. In males, the finding implies a somewhat negative linear relationship “at first” with higher BMI or WC associated with lower VSR. While a BMI > 25 is associated with a greater risk of obesity-related illnesses and mortality [1], which are likely driven by an increase in total visceral fat mass, these changes are not consistent with the changes seen in fat distribution between the visceral cavity and the more innocuous subcutaneous regions. It could be that there is a change in the relative distribution of abdominal fat with increasing BMI or WC, but clearly, the slope flattens out around BMI = 30 kg/m^2^. When the BMI or WC reaches the inflection point (BMI = 39.08 kg/m^2^; WC = 144.5 cm), the continued increase in BMI or WC results in a greater VSR. For women, these relationships appear to follow the opposite pattern, but there was considerably less variation in women than in men. That is, for females with a BMI or WC lower than 38.75 kg/m^2^ and 43.5 cm, respectively, there is a positive relationship between these parameters and VSR. However, such a relationship is reversed when BMI or WC reaches 38.75 kg/m^2^ and 43.5 cm, respectively, or higher. Studies on the relationship between BMI, WC and VSR are limited. One study reported that the relationships between BMI or WC and VSR were positive in women, whereas they were negative in men [19], which is different from the concave and convex relationships observed between BMI or WC and VSR for male and female individuals, respectively, in the current study. The discrepancy between previous findings and ours could be explained by the different sample age ranges (current study: 20–59 years vs. other studies ≥ 35 years) and geographic and sample sizes (this study: national, *n* = 11,943 vs. other studies: regional, *n* = 3223). Given the representation of our sample, the current study offers important information regarding the relationships of anthropometric parameters with VSR. Our findings indicate that male individuals presenting a high BMI/WC, who might seem underweight or have BMI ≥ 40 kg/m^2^ [34], have a greater risk for obesity-related illness than BMI/WC represents. In contrast, females with a BMI ≥ 40 kg/m^2^ have a lower risk than previously reported. Our findings indicate that using BMI or WC alone might not be optimal for assessing health risks. Therefore, further research is warranted to explore more accurate prediction of VSR using anthropometric measures, which are practical and economical, to help health practitioners better address body-fat-distribution-related health risks in public health.

## 5. Strengths and Limitations

The present study has strengths and limitations. The major strengths of the study are that (1) it is the first to utilize abdominal-related measures derived from DXA, a two-dimensional imaging technique for adipose tissue assessment, for a large national representative adult sample including multiple racial and ethnic groups. Therefore, our findings are more generalizable for public health purposes than previous studies. (2) Height, weight, WC and DXA scans were obtained using standardized procedures by trained research staff, ensuring consistency throughout the cohort used for our analysis [22,23]. Among the limitations, the study cohort was limited to individuals aged between 20 and 59 years, which included the full set of parameters selected for our analysis. Secondly, menopausal status was not considered, which could influence the distribution of visceral and subcutaneous fat in postmenopausal women [35]. Lastly, we are also limited by the DXA scan exclusion criteria, such as possible or ongoing pregnancy, and the size of the DXA table, which is limited to respondents less than 450 pounds and less than 6′5″ [23].

## 6. Conclusions

In conclusion, we found positive relationships between anthropometric measures and VFA, but Whites had higher slopes than Blacks, Hispanics and/or Asians, and Black had lower slope parameters than Hispanics and Asians. The relationship between anthropometric measures and VSR was concave in males and convex in females. This finding reflects the sex difference in fat distribution in relation to BMI and WC. These relationships should be taken into consideration while working with different racial/ethnic groups addressing fat-distribution-related health concerns.

## Figures and Tables

**Table 1:** Demographics characteristics stratified by sex, NHANES 2011–2018.

Variables	Total	Male	Female	*p* Values
	*n* = 11,943	6049 (51.6%)	5894 (48.4%)	
Age (years)	39.69 ± 0.23	39.33 ± 0.22	40.08 ± 0.30	<0.001 *
Race/ethnicity, *n* (weighted %)				
White	4161 (61.7)	2144 (61.6)	2017 (61.8)	0.768
Black	2671 (11.8)	1322 (11.3)	1349 (12.4)	0.01 *
Hispanic	2896 (17.0)	1416 (17.7)	1480 (16.3)	0.003 *
Asian	1695 (5.8)	881 (5.6)	814 (5.9)	0.164
Others	520 (3.8)	286 (3.9)	234 (3.6)	0.589
Education, *n* (weighted %)				
High school or less	4789 (34.7)	2674 (38.7)	2115 (30.4)	<0.001 *
Some college or more	7152 (65.3)	3375 (61.3)	3777 (69.6)	<0.001 *
Ratio of family income to poverty, *n* (weighted %)				
<1.0	2495 (15.8)	1189 (14.7)	1306 (17.1)	<0.001 *
≥1.0	8465 (84.2)	4342 (85.3)	4123 (82.9)	<0.001 *
Body mass index (kg/m^2^)	29.01 ± 0.13	28.81 ± 0.14	29.23 ± 0.18	<0.001 *
Weight status, *n* (weighted %)				
Underweight	214 (1.5)	93 (1.2)	121 (1.9)	0.004 *
Normal	3402 (28.2)	1654 (25.1)	1748 (31.6)	<0.001 *
Overweight	3721 (32.0)	2169 (36.5)	1552 (27.2)	<0.001 *
Obese	4532 (37.6)	2095 (36.6)	2437 (38.7)	0.11
Waist circumference (cm)	98.27 ± 0.33	100.26 ± 0.37	96.14 ± 0.43	<0.001 *
Abdominal obesity, *n* (weighted %)	6082 (52.6)	2327 (42.2)	3755 (63.8)	<0.001 *
VFA (cm^2^)	105.68 ± 1.19	112.79 ± 1.36	98.09 ± 1.47	<0.001 *
VSR	0.35 ± 0.00	0.45 ± 0.00	0.24 ± 0.00	<0.001 *

Note: Data are presented as weighted mean ± standard errors unless otherwise specified. *p*-values for continuous variables were obtained by performing *t*-tests (PROC SURVEYREG), and *p*-values for categorical variables were obtained by performing Chisq-test (PROC SURVEYFREQ) in SAS. VFA = visceral fat area, VSR = the ratio of visceral to subcutaneous adipose area, * symbol indicated statistical significance.

**Table 2:** The relationships between BMI, WC and VFA (cm^2^).

	Overall			Male			Female		
	Adj. β (95% CI)	*p*-Value	R-Square	Adj. β (95% CI)	*p*-Value	R-Square	Adj. β (95% CI)	*p*-Value	R-Square
**BMI**									
Total	5.86 (5.56, 6.15)	<0.001 *	0.609	6.12 (5.74, 6.51)	<0.001 *	0.598	5.67 (5.32, 6.01)	<0.001 *	0.619
White	6.40 (6.02, 6.78) ^abc^	<0.001 *	0.633	6.70 (6.14, 7.26) ^abc^	<0.001 *	0.603	6.18 (5.73, 6.63) ^abc^	<0.001 *	0.654
Black	3.79 (3.42, 4.16) ^adef^	<0.001 *	0.508	4.24 (3.79, 4.70) ^adef^	<0.001 *	0.56	3.51 (3.09, 3.93) ^adef^	<0.001 *	0.471
Hispanic	5.63 (5.19, 6.08) ^bd^	<0.001 *	0.570	5.66 (4.91, 6.41) ^bd^	<0.001 *	0.549	5.62 (5.17, 6.07) ^bdg^	<0.001 *	0.589
Asian	6.04 (5.43, 6.64) ^e^	<0.001 *	0.589	5.40 (4.56, 6.25) ^ce^	<0.001 *	0.495	6.59 (5.97, 7.22) ^egh^	<0.001 *	0.657
Others	5.51 (5.11, 5.91) ^cf^	<0.001 *	0.641	5.79 (5.13, 6.44) ^f^	<0.001 *	0.664	5.34 (4.70, 5.99) ^cfh^	<0.001 *	0.628
*p*-value for interaction term (BMI × race)	<0.001 *			<0.001 *			<0.001 *		
**WC**									
Total	2.64 (2.53, 2.75)	<0.001 *	0.684	2.60 (2.48, 2.72)	<0.001 *	0.673	2.67 (2.53, 2.80)	<0.001 *	0.690
White	2.85 (2.72, 2.99) ^abcd^	<0.001 *	0.710	2.84 (2.67, 3.01) ^abcd^	<0.001 *	0.684	2.85 (2.69, 3.01) ^a^	<0.001 *	0.723
Black	1.75 (1.61, 1.89) ^aefg^	<0.001 *	0.583	1.77 (1.61, 1.93) ^aefg^	<0.001 *	0.633	1.73 (1.56, 1.91) ^bcd^	<0.001 *	0.538
Hispanic	2.64 (2.50, 2.79) ^be^	<0.001 *	0.657	2.52 (2.29, 2.76) ^be^	<0.001 *	0.635	2.75 (2.58, 2.91) ^ab^	<0.001 *	0.677
Asian	2.67 (2.46, 2.87) ^cf^	<0.001 *	0.678	2.41 (2.13, 2.69) ^cf^	<0.001 *	0.625	2.94 (2.69, 3.19) ^ce^	<0.001 *	0.714
Others	2.46 (2.25, 2.68) ^dg^	<0.001 *	0.677	2.35 (2.03, 2.67) ^dg^	<0.001 *	0.696	2.60 (2.28, 2.92) ^de^	<0.001 *	0.661
*p*-value for interaction term (WC × race)	<0.001 *			<0.001 *			<0.001 *		

Note: A linear regression model was used to estimate the association between BMI, WC and VFA adjusted for age, sex, race/ethnicity, education, and the ratio of family income to poverty; letters a–h indicate racial/ethnic group differences, and if there are two groups with the same letter then these two groups are different statistically; BMI = body mass index, WC = waist circumference, VFA = visceral fat area; * symbol indicates statistical significance.

**Table 3:** The relationships between BMI, WC and VSR.

	Male					Female				
	Adj. β1 (95% CI) (Linear)	*p*-Value (Linear)	Adj. β2 (95% CI) (Quadratic)	*p*-Value (Quadratic)	R-Square	Adj. β1 (95% CI) (Linear)	*p*-Value (Linear)	Adj. β2 (95% CI) (Quadratic)	*p*-Value (Quadratic)	R-Square
**BMI**										
Total	−0.0469 (−0.0523, −0.0414)	<0.001 *	0.0006 (0.0005, 0.0006)	<0.001 *	0.308	0.0155 (0.0130, 0.0180)	<0.001 *	−0.0002 (−0.0002, −0.0001)	<0.001 *	0.290
White	−0.0400 (−0.0484, −0.0316) ^a^	<0.001 *	0.0005 (0.0003, 0.0006) ^a^	<0.001 *	0.272	0.0152 (0.0113, 0.0191) ^a^	<0.001 *	−0.0002 (−0.0002, −0.0001) ^a^	<0.001 *	0.289
Black	−0.0899 (−0.1001, −0.0797) ^abcd^	<0.001 *	0.0011 (0.0009, 0.0012) ^abcd^	<0.001 *	0.531	0.0055 (0.0015, 0.0096) ^abcd^	0.008 *	−0.0001 (−0.0001, −0.0000) ^abc^	0.006 *	0.166
Hispanic	−0.0335 (−0.0455, −0.0215) ^be^	<0.001 *	0.0004 (0.0002, 0.0005) ^be^	<0.001 *	0.327	0.0200 (0.0155, 0.0246) ^b^	<0.001 *	−0.0003 (−0.0003, −0.0002) ^b^	<0.001 *	0.286
Asian	−0.0402 (−0.0538, −0.0266) ^cf^	<0.001 *	0.0005 (0.0003, 0.0007) ^c^	<0.001 *	0.342	0.0201 (0.0093, 0.0309) ^c^	<0.001 *	−0.0003 (−0.0005, −0.0001) ^c^	0.013 *	0.331
Others	−0.0643 (−0.0810, −0.0476) ^def^	<0.001 *	0.0008 (0.0006, 0.0011) ^de^	<0.001 *	0.453	0.0150 (0.0045, 0.0254) ^d^	0.006 *	−0.0002 (−0.0003, −0.0001)	0.014 *	0.244
*p*-value for interaction term (BMI × race)	<0.001 *		<0.001 *			<0.001 *		<0.001 *		
**WC**										
Total	−0.0289 (−0.0322, −0.0256)	<0.001 *	0.0001 (0.0001, 0.0001)	<0.001 *	0.331	0.0087 (0.0073, 0.0101)	<0.001 *	−0.0001 (−0.0001, −0.0000)	<0.001 *	0.316
White	−0.0254 (−0.0307, −0.0201) ^a^	<0.001 *	0.0001 (0.0001, 0.0001) ^a^	<0.001 *	0.286	0.0087 (0.0067, 0.0106) ^a^	<0.001 *	−0.0001 (−0.0001, −0.0000)	<0.001 *	0.326
Black	−0.0456 (−0.0504, −0.0408) ^abcd^	<0.001 *	0.0002 (0.0002, 0.0002) ^abcd^	<0.001 *	0.575	0.0041 (0.0008, 0.0074) ^abcd^	0.014 *	−0.0001 (−0.0001, −0.0000) ^bcd^	0.019 *	0.167
Hispanic	−0.0192 (−0.0257, −0.0127) ^be^	<0.001 *	0.0001 (0.0000, 0.0001) ^be^	<0.001 *	0.330	0.0107 (0.0079, 0.0135) ^b^	<0.001 *	−0.0001 (−0.0001, −0.0000) ^b^	<0.001 *	0.298
Asian	−0.0235 (−0.0322, −0.0149) ^c^	<0.001 *	0.0001 (0.0001, 0.0001) ^c^	<0.001 *	0.348	0.0126 (0.0081, 0.0171) ^c^	<0.001 *	−0.0001 (−0.0001, −0.0000) ^c^	<0.001 *	0.359
Others	−0.0332 (−0.0403, −0.0260) ^de^	<0.001 *	0.0001 (0.0001, 0.0002) ^de^	<0.001 *	0.482	0.0108 (0.0032, 0.0184) ^b^	0.006 *	−0.0000 (−0.0001, −0.0000) ^b^	0.013 *	0.280
*p*-value for interaction term (WC × race)	<0.001 *		<0.001 *			<0.001 *		<0.001 *		

Note: A polynomial linear regression model with quadratic terms was used to estimate the association between BMI or WC with VSR adjusted for age, sex, race/ethnicity, education and the ratio of family income to poverty; letters a–f indicate racial/ethnic group differences, and if there are two groups with the same letter then these two groups are different statistically; BMI = body mass index, WC = waist circumference, VFA = visceral fat area; * symbol indicates statistical significance.

## Data Availability

These publicly accessible data were available on Centers for Disease Control website: https://wwwn.cdc.gov/nchs/nhanes/sasviewer.aspx, accessed on 16 June 2022.
